# Case Report: Novel molecular characterization of rare primary mucinous adenocarcinoma of the renal pelvis

**DOI:** 10.3389/fonc.2025.1678970

**Published:** 2026-01-12

**Authors:** Megan K. Taylor, Irasema C. Paster, Dory E. Arevalo Salazar, Juan Chipollini, Alejandro Recio Boiles

**Affiliations:** Department of Medicine, The University of Arizona College of Medicine - Tucson, Tucson, AZ, United States

**Keywords:** biomarker, case report, genomic, kidney cancer, mucinous

## Abstract

Mucinous adenocarcinoma of the renal pelvis (MAC-RP) is an exceedingly rare condition, accounting for less than 1% of malignancies originating from this epithelial site. We report a case of MAC-RP in a woman presenting with right flank pain from obstructing stones, recurrent urinary tract infections, and ureteral stents. Imaging showed a right kidney mass consistent with xanthogranulomatous pyelonephritis (XGP) with an abscess surgically removed. Pathology after radical nephrectomy unexpectedly revealed *in situ* MAC-RP with intestinal metaplasia at the margin. Surveillance testing showed positive levels of CEA, CA19-9, and novel ctDNA and cfDNA despite negative results from pan-endoscopy and imaging. A subsequent monitoring imaging identified a right flank mass that was resected, confirming recurrent MAC-RP. The patient completed 6 months of adjuvant mFOLFOX6, as recommended in the MAC-colon cancer guidelines. The patient completed the treatment without any evidence of measurable molecular or radiological disease. At 4 months after the last systemic therapy, the patient’s biomarkers were rising, matching radiological local, ovarian, and pleural recurrences, which were histologically confirmed. Since there are no established guidelines for MAC-RP, our team is the first to apply cutting-edge cancer technologies to inform future clinical decisions and molecular tumor board discussions and to compare the genomic distance of MAC-RP with that of other bladder or colorectal origin sites.

## Introduction

Primary renal pelvis adenocarcinoma is rare, making up less than 1% of malignancies originating from the renal pelvis epithelium, with less than 100 cases documented in the literature (1, 2). The few cases reported in the literature show similar predisposing factors, such as chronic nephrolithiasis and recurrent urinary tract infections, which lead to inflammation and glandular metaplasia. Xanthogranulomatous pyelonephritis (XGP) is a rare variant of chronic pyelonephritis (3). It involves chronic granulomatous inflammation marked by lipid-laden macrophages replacing renal parenchyma, suspected to result from recurrent bacterial infections and an immune response (4). While renal cell carcinoma is the most common malignancy associated with XGP, squamous cell carcinoma and rarely adenocarcinoma of the renal pelvis have also been reported (5). We present the molecular tissue and blood profiling of a rare case of primary MAC-RP with XGP. We aim to support treatment decisions in situations where there is no clear consensus among guidelines or expert opinions.

## Patient’s information

An African-American woman in her late 60s presented to the emergency department (ED) with right flank pain lasting 2 days. She denied hematuria, dysuria, or pyuria. Her past medical history included recurrent obstructive nephrolithiasis with urinary tract infections due to staghorn calculi (approximately 50 episodes since age 11), leading to obstructive hydronephrosis, chronic kidney disease (CKD) stage IV, and severe iron deficiency anemia. She had a prior parathyroid adenoma removed, and previous screenings for breast, cervical, and colorectal cancer were negative. History was significant for 10 family members, mostly in their early 50s, with brain, kidney, stomach, prostate, and breast cancer, but she had no knowledge of hereditary cancer syndrome.

### Clinical findings

**Figure 1:**
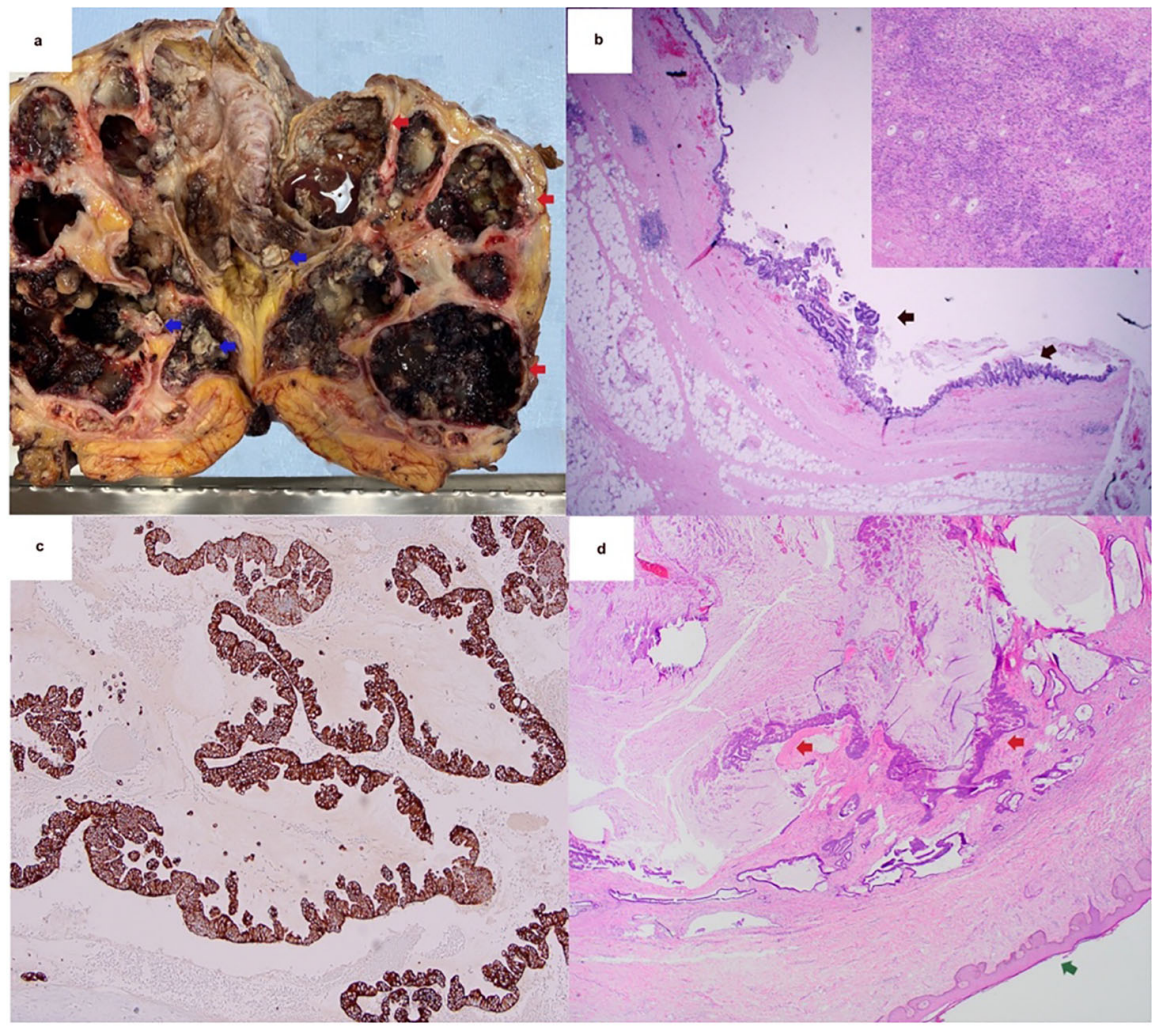
Preoperative radiological findings: MRI on initial workup showed a massively enlarged right kidney with classic features of xanthogranulomatous pyelonephritis. Severe right hydroureteronephrosis is present secondary to staghorn calculi, with debris within the collecting system. Final image showing a 7.2 x 2.4-cm soft tissue collection along the inferior pole of the right kidney. Postoperative pathology: **(a)** Gross picture of the right kidney showing dilated calyces and dilated renal pelvis (red arrows). The renal pelvis is filled with yellow-tan purulent material admired with hemorrhage and associated multiple yellow-tan calculi (blue arrows). **(b)** A representative section of the renal pelvis showing strips of malignant-appearing glandular epithelium replacing the normal urothelium lining of the renal pelvis floating with abundant mucin (black arrows). Moreover, in the right upper corner is a close-up of the dense inflammatory component, consisting of lymphocytes, plasma cells, neutrophils, and histiocytes admixed with seldom tubules (H&E stain, ×20). **(c)** Malignant glandular cells strongly positive to the cytokeratin 7 (CK7) immunohistochemical stain. GATA3 is focally positive (not shown), CDX2 is patchy positive (not shown), and SATB2 is negative (not shown). These findings are consistent with adenocarcinoma of the renal pelvis with mucinous features. **(d)** Representative section of a later excision of the right flank and rib, consisting of malignant glandular cells similar to the one found on the kidney, involving the dermis (red arrows). Epidermis, green arrow (H&E stain, ×20).

On ED evaluation, computed tomography (CT) of the abdomen and pelvis without contrast due to severe CKD (eGFR 12 mL/min) showed severe right hydroureteronephrosis, numerous cysts replacing the right parenchyma, and a large obstructing staghorn calculus. A soft tissue collection at the inferior pole raised concern for abscess or mass. MRI confirmed findings consistent with XGP, which was later complicated by a painful, purulent right flank nephrostomy tube with cutaneous fistula, prompting a right radical nephrectomy. Pathology confirmed XGP and unexpectedly revealed *in situ* mucinous adenocarcinoma of the renal pelvis with intestinal metaplasia at the negative margins (**Figure 1**). The patient was discharged to be followed by Medical Oncology for rare malignancy.

### Therapeutic intervention

The Genitourinary Multidisciplinary Tumor Board (GU MTB) discussion recommended surveillance due to a lack of evidence supporting adjuvant chemotherapy for non-invasive mucinous adenocarcinoma pathologic tumor *in situ* (pTis) with moderately differentiated grade 2 and negative margins (6). This was extrapolated from bladder and colorectal guidelines (7, 8). Recommendations were made to follow up post-operatively with gastrointestinal (GI) tumor markers carcinoembryonic antigen (CEA) and cancer-associated antigen 19-9 (CA19-9) and close monitoring imaging by whole-body positron emission tomography (PET/CT) and to add novel circulating tumor DNA (ctDNA by Signatera^®^).

**Figure 2:**
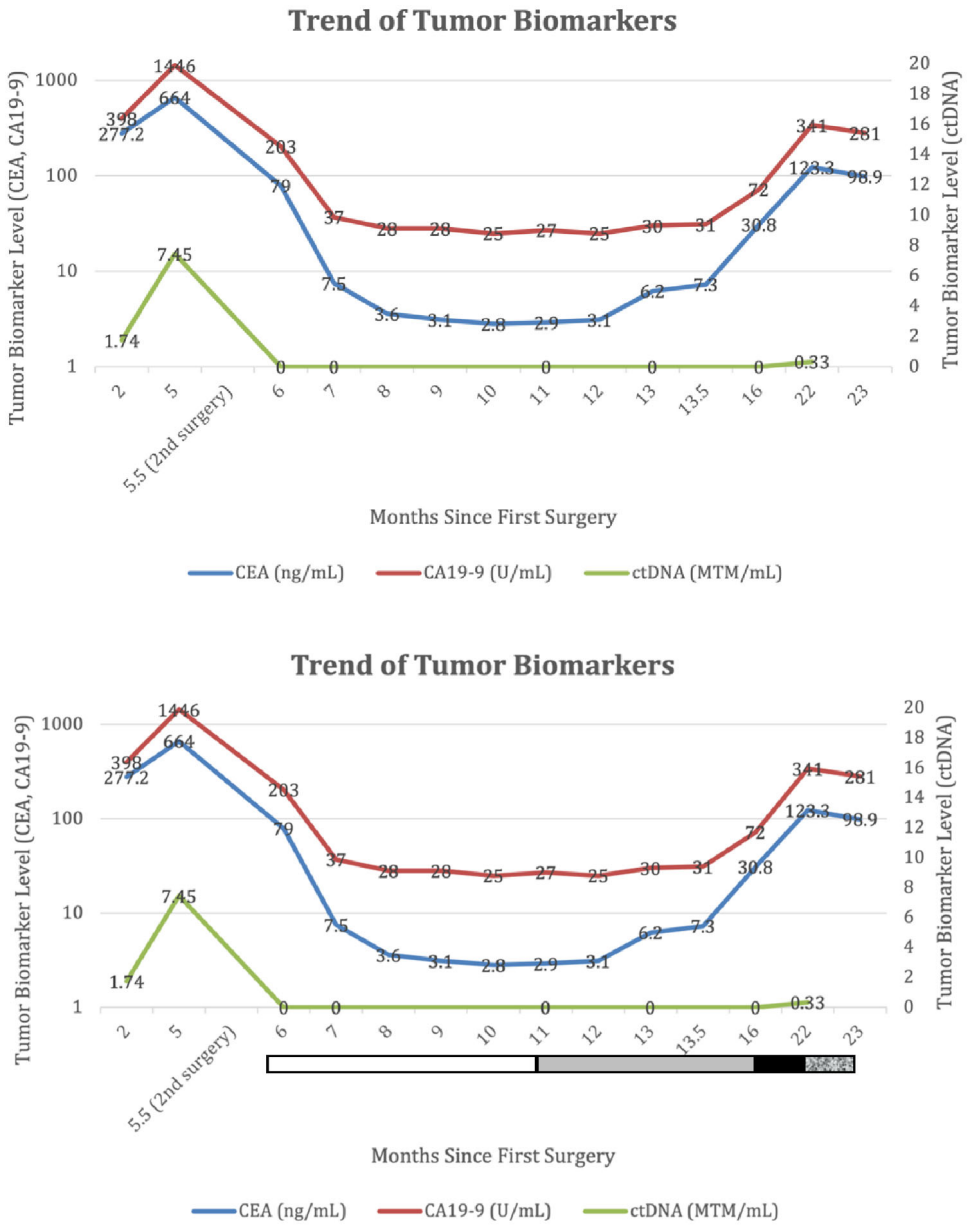
Line graph showing trends of tumor biomarkers, including CEA and CA19-9 (left y-axis) and ctDNA (right y-axis). The x-axis indicates months since the patient’s first surgery (right radical nephrectomy). CEA, carcinoembryonic antigen; CA19-9, carbohydrate antigen 19-9; ctDNA, circulating tumor DNA. The white-filled box indicates 6 months of adjuvant mFOLFOx, the gray-filled box shows 4 months of surveillance, and the black-filled box represents 6 months of follow-up. The gray-gradient box illustrates the start and continuation of Carbo-Gem-Nivo.

On follow-up, ctDNA and tumor markers were immediately positive for suspected residual and recurrent disease (CEA—277.2 ng/mL, CA19-9—398 U/mL, and ctDNA—1.74 MtM/mL) (**Figure 2**); however, PET/CT and MRI showed nonspecific surgical findings. At 2 months later, PET/CT showed local recurrence at the previous fistula site. At the second GU MTB, it was recommended to pursue salvage surgery of an aggressive malignancy followed by adjuvant systemic therapy. A right flank mass and rib excision confirmed metastatic mucinous adenocarcinoma with dermal and rib involvement and focally positive margins. The patient was treated with 6 months of mFOLFOX6, modeled after colorectal cancer guidelines due to the absence of renal-specific protocols.

### Diagnostic assessment

**Figure 3:**
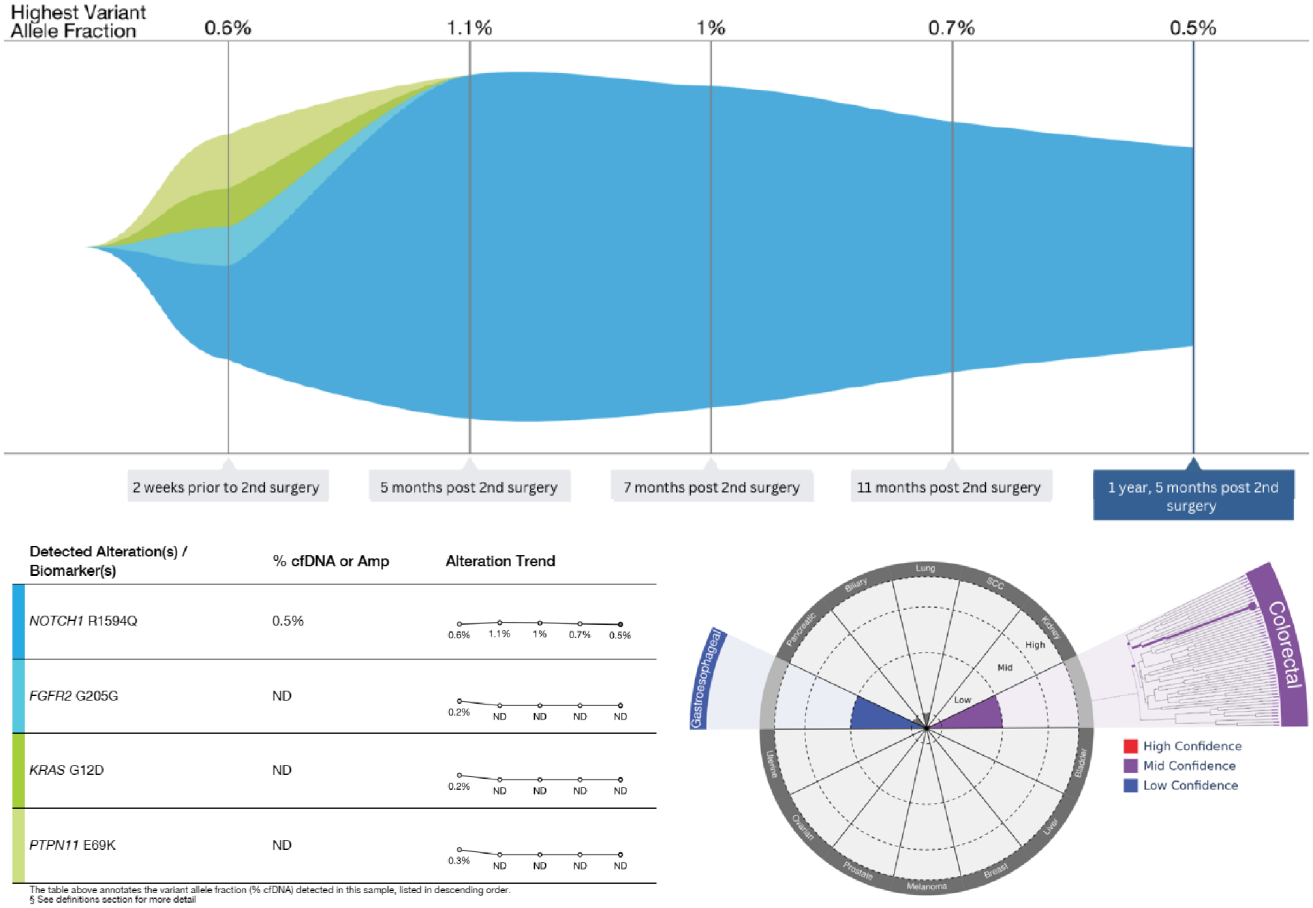
Tumor mutation response of liquid biopsies by Guardant360^®^. %cfDNA, percent of cell-free DNA. Molecular prediction of tumor type and/or subtype based on DNA methylation signatures is provided as a supplement, not a replacement, for standard clinical and pathologic evaluation.

Medical Oncology decided to investigate this rare cancer through molecular profiling of blood and tissue. Caris^®^ profiling of DNA, RNA, and proteins from the primary renal pelvis reported pathogenic variants KRAS[G12D], U2AF1[S34F], ERBB2[S310F], and FBXW7[W244], compared to those from the recurrent flank mass with KRAS[G12D], MSH3[c.1341-1G>T], and U2AF1[S34F], with other pertinent negatives like proficient MMR, TMB low, MSS, PDL1 CPS 0, and previously positive ERBB2 and FBXW7 not seen on recurrent metastatic mass (**Table 1**). Liquid biopsy (Guardant360^®^) revealed NOTCH1[R1594Q] (0.6%), PTPN11[E69K] (0.3%), KRAS[G12D] (0.2%), and FGFR2[G205G] (0.2%) mutations on cell-free DNA (cfDNA) (**Table 1**, **Figure 3**). Furthermore, liquid biopsy molecular tumor profiling had a primary prediction (colorectal, 50%) and secondary prediction (gastroesophageal, 50%). Genetic counseling revealed MSH3 pathogenic variant c.1341-1G>T, considered autosomal recessive.

**Table 1:** Comprehensive molecular profiling of DNA, RNA, and IHC protein of the primary renal pelvis by Signatera^®^, liquid biopsy analysis by Guardant360^®^, recurrent metastatic flank mass by Caris^®^, and germline testing by Ambry Genetic^®^.

Specimen	Detected alteration(s)/biomarker(s)	Biopsy method	Result
Primary renal pelvis carcinoma *in situ* specimen (first surgery D0)	KRAS	Tissue	Pathogenic variant G12D
	U2AF1^a^	Tissue	Pathogenic variant S34F
	ERBB2	Tissue	Pathogenic variant S310F
	FBXW7^a^	Tissue	Pathogenic variant W244
Blood cfDNA (5 months)	NOTCH1^b^	Liquid	R1594Q (0.6%)
	PTPN11^b^	Liquid	E69K (0.3%)
	KRAS	Liquid	G12D (0.2%)
	FGFR2^b^	Liquid	G205G (0.2%)
Metastatic recurrent flank mass (salvage surgery)	KRAS	Tissue	Pathogenic variant G12D
	MSH3^c^	Tissue	Pathogenic variant c.1341-1G>T
	U2AF1^a^	Tissue	Pathogenic variant S34F
	ERBB2	Tissue	Negative
	FBXW27^a^	Tissue	Negative
All samples	MMR	Tissue	Proficient
	TMB	Tissue/liquid	Low
	MSI	Tissue/liquid	Stable
	PD-L1	Tissue	Negative
Germline testing	MSH3^c^	NA	Pathogenic variant c.1341-1G>T (autosomal recessive)

MMR, mismatch repair; TMB, tumor mutational burden; MSI, microsatellite instability; PDL1, programmed death receptor ligand-1; cfDNA, cell-free DNA.

a The selected panel of biomarkers by liquid biopsy did not include U2AF1 or FVXW7.

b NOTCH, PTPN11, and FGFR2 were found in tissue but considered to be clonal hematopoiesis of indeterminate potential (CHIP) by CARIS.

c For tissue biopsy data, MMR status was assessed by immunohistochemical (IHC) analysis. The antibodies used were MLH1 (M1), MSH2 (G2191129), MSH6 (44), and PMS2 (EPR3947). Deficiency/loss of nuclear expression of any of the MMR proteins (MLH1, MSH2, MSH6, and PMS2) results in mismatch repair deficiency (loss). Intact nuclear expression of all the MMR proteins is considered mismatch repair proficient (intact).

### Follow-up and outcome

After two chemotherapy cycles, the tumor markers normalized (CEA 3.6 ng/mL and CA19-9–28 U/mL) (**Figure 2**). At the 3-month mid-regimen evaluation, ctDNA was undetectable, and the liquid biopsy mutations showed a molecular mutation response with only NOTCH1 detectable, considered a clonal hematopoiesis of indeterminate potential (CHIP) mutation. These results were deemed consistent with the MRI findings of treatment response. At the end of the 6-month adjuvant therapy, although the tumor markers were rising, they remained within normal limits; ctDNA was still undetectable, and there was no measurable disease on imaging. The patient tolerated chemotherapy well, with no major adverse events, and a decision was made to continue close surveillance.

Concurrently with our case report, we genomically compared MAC-RP to other bladder or colorectal origin sites using three techniques (9): DNA sequencing, RNA sequencing (WTS), and immunohistochemistry (IHC) protein analysis with varying measures of gene expression at Caris Life Sciences. Our null hypothesis was that mucinous adenocarcinomas of the urothelial sites are genomically closer to a urothelial origin; conversely, the alternative hypothesis is that they are closer to a colonic origin. Histopathology was used to categorize five cancer site groups: (1) non-mucinous (non-MUC), non-adenocarcinoma (non-AC) urinary carcinoma (UC) arising in the bladder, ureter, urethra, or renal pelvis with any transitional cell carcinoma (TCC) UC-nonMUC-nonAC[N156 samples], (2) UC-nonMUC-AC[N83], (3) UC-MUC-AC[N16], (4) CRC-MUC-AC[N3100], and (5) CRC-nonMUC-AC[N38205]. The primary aim was to determine which other group was closest to group 3, our control. The secondary aim was to determine if group 2 was genetically closer to group 1 or group 5. Only genes with a positive expression were included in the cluster analysis. ANOVA analysis was conducted to explore the relationships between the groups further. A log transformation was performed to achieve normality. To further investigate pairwise comparisons, a *post hoc* Dunnett test was employed.

In our initial analysis, the group 3 gene expression ratio was predominantly associated with UC groups 1 and 2 (24 of 33) and, to a lesser extent, with CRC groups 4 and 5 (nine of 33) (all *p*-values >0.05) (9). In the second analysis, the overall ANOVA model comparing the gene expression between histological groups 1, 2, and 5 was statistically significant (*p* = 0.0018). The Dunnett test revealed a statistically significant difference between group 2 and group 5 (p-value = 0.001). However, the closest difference was between group 2 and group 1 (*p* = 0.100).

At 4 months post-adjuvant therapy, the tumor markers rose (CEA 30.8 ng/mL and CA19-9–72 U/mL), possibly due to chronic xanthogranulomatous inflammation of the left chronic kidney disease XGP, but ctDNA and cfDNA remained undetectable, and no residual avid disease was detected on imaging at this time. Unfortunately, the patient became homeless and was lost to follow-up for 6 months. Upon return, imaging revealed a persistent surgical bed mass of 1.5 × 1.0 cm, a new complex left ovarian cyst 3.5 × 2.3 cm, and pleural abnormalities. A left ovary needle biopsy and pleural cytology confirmed adenocarcinoma with mucinous features consistent with the previous adenocarcinoma of the renal pelvis. The tumor markers rose: CEA, 123.3 ng/mL; CA19-9, 341 U/mL; and ctDNA, 0.33 MtM/mL. However, no pathogenic mutation-positive cfDNA was detected.

Her case was presented at the Caris MTB™. A trial targeting KRAS G12D or U2AF1 was advised, but the patient was ineligible due to CKD and anemia. NGS of ovarian biopsy was recommended. As a non-cisplatin candidate with a UC-related genomic profile, the patient began combined immunotherapy (nivolumab) and chemotherapy (platinum-based regimen) as per the Urothelial Carcinoma and CheckMate 901 trial guidelines, allowing cisplatin to be switched to gemcitabine–carboplatin for up to six cycles, followed by immunotherapy. The initial biomarker response showed reductions (CEA 69.7 ng/mL, CA19-9–270 U/mL, and ctDNA 0.15 MtM/mL); the cfDNA mutations remained undetectable, and imaging revealed a partial response. The patient remains alive over 40 months post-diagnosis.

### Timeline of tumor biomarkers

For details on the timeline of tumor biomarkers, see **Figure 2**.

## Discussion

### Diagnostic challenges

The clinical manifestations of MAC-RP vary and can be nonspecific, with some patients reporting no symptoms and others experiencing abdominal mass, hematuria, or flank pain (1). Primary renal adenocarcinoma is historically difficult to diagnose, mainly due to its appearance on imaging, and no standard surgical procedure has been established; most patients undergo treatment with radical nephrectomy (1). MAC usually occurs in the ovarian and colorectal regions (1). Therefore, metastases of adenocarcinoma from other organs should be investigated first using imaging and gastrointestinal endoscopy. No guidelines or evidence support adjuvant systemic cancer therapy for MAC-RP, although, given the high risk of recurrence, this is standard practice for MAC of the ovary and colon.

### Biomarkers’ interpretation

Additionally, there are no reports on MAC-RP providing detailed molecular profiling to better guide treatment decisions. Zhang et al. (2024) (10) reported only for KRAS (Kirsten rat sarcoma viral oncogene homolog) mutation on three cases of MUC-RP, with only one patient harboring a pathogenic mutation G12D, as our patient had. The real frequency of the KRAS mutation in MAC-RP remains unknown compared to CRC, which has a high rate of 45% activating mutations, but is significantly lower at 11% in bladder adenocarcinoma (11). U2AF1 (U2 small nuclear RNA auxiliary factor 1) mutations are infrequent in solid malignancies, whether from the bladder or colon, and are typically passenger mutations or occur in the context of hypermutated tumors. FBXW7 (F-box and WD repeat domain-containing 7) occurs in approximately 8% of CRCs (12), making it the second most commonly mutated ubiquitin ligase gene after APC, often co-occurring with KRAS, and carries a poor prognosis, including chemoresistance to platinum, as similarly reported in bladder cancer, but at a lower rate of 6% (13). The significance of the first test, ERBB2/HER2 in our case, remains unknown as the mutation has not been confirmed in later studies. With a noticeable family history prompting the need for genetic counseling, the germline MSH3 (MutS homolog 3) mutation alone may only cause a partial MMR deficiency; IHC proved to be proficient.

### Genomic classification and treatment implications

Notably, mucinous adenocarcinomas of the bladder with urothelial origin (MAC-UC) bear a histopathological resemblance to intestinal tumors, constituting the third predominant subtype after classical transitional cell (TCC) and squamous cell carcinomas (11). Alternatively, treatment decisions based on gene expression similarities, rather than histological similarities, are an emerging concept in rare cancers. This is an unexplored area in MAC-RP treatment, prompting our investigation into genomic comparisons with MAC-UC and mucinous colorectal adenocarcinoma (MAC-CRC). In our study, we hypothesize that an all-encompassing genomic profile may reveal analogous biomarkers that are more closely associated with the MAC-CRC than the MAC-UC origin site, thereby providing better guidance for the management of this rare cancer, as extrapolated from respective guidelines. We showcase this concept in our case report of a patient with MAC-RP. Our genomic comparison (9), which examined the proximity of the UC-MUC-AC group (due to a lack of cases specifically for MAC-RP, serving as a surrogate control), revealed that it was closer to groups 1 and 2 of urothelial origin and farther away from groups 4 and 5 of colon origin. Furthermore, we noticed that group 2, urothelial adenocarcinoma, was closer to group 1, urothelial carcinoma, and farther from 5roup 5, colorectal carcinoma, while controlling for non-mucinous tumors within each group. Suppose this concept is genuine, it translates to RP-MAC being closer to urothelial origin than colorectal, which would require further research. In that case, the current proposal is to treat urothelial adenocarcinomas with or without mucinous features according to urothelial carcinoma guidelines rather than relying on colorectal expert opinion consensus extrapolated recommendations.

### Implications for future research

CEA and CA19–9 were selected based on MAC-RP case reports involving 20% of patients and extrapolated from GI malignancies, which have a 70%–80% positive rate and share a similar histology (1, 14). However, in both MAC-RP and GI cancers, CEA and CA19–9 may not always be present or of clinical relevance. Chronic xanthogranulomatous inflammation can drive elevated CEA and CA 19–9 through reactive epithelial secretion and tissue destruction, and marker normalization following resection supports an inflammatory rather than malignant origin. Therefore, novel technologies adapted from evidence in bladder and colon cancer, utilizing ctDNA and cfDNA monitoring for minimal residual disease and tumor response, respectively, were successfully adopted in this case (15, 16). Future patient care discussions should include the benefits of early detection of molecular residual disease against the risk of a potential non-diagnostic imaging, with a time lead of 10 months for CRC and 4 months for UCa (17), to inform decisions on the initiation of systemic therapy. Our patient had positive CEA, CA19-9, and ctDNA levels before the first radiological recurrence. Later, they demonstrated measurable molecular and radiological clearance with adjuvant treatment. During surveillance, the earlier elevations of CEA and CA19-9, possibly attributed to chronic xanthogranulomatous inflammation, compared with ctDNA are likely due to fundamental differences in biomarker biology. CEA and CA19–9 are actively secreted by viable, glandular-differentiated tumor cells. In contrast, ctDNA detection depends on tumor cell turnover and DNA shedding, which may occur later and require sufficient tumor burden. Tumors may secrete markers before sufficient apoptotic/necrotic tumor turnover generates detectable ctDNA, therefore offering complementary clinical information. A noticeable uptrend correlating with disseminated metastatic disease continued to show guidance benefit with frontline systemic therapy, making this a reliable biomarker. Unfortunately, cfDNA by liquid biopsy was not considered a reliable biomarker due to its multiple limitations; the pre-selected panel of biomarkers by liquid biopsy did not include U2AF1 or FBXW7, loss of reliable mutation tracking after loss of KRAS, not considering clonal hematopoiesis of indeterminate potential (CHIP) such as NOTCH, PTPN11, and FGFR2, and moderate confidence on the molecular prediction of tumor type suggesting CRC origin. We acknowledge that cfDNA limitations are tumor-dependent, as not all tumors shed cfDNA equally, and that the origin of the DNA fragments is not under control, which can be somatic tumor, germline, or CHIP. In our cfDNA context, several mutation-positive findings were consistent with CHIP, reflecting age-related clonal hematopoiesis rather than true tumor-derived alterations. The risk of false tumor attribution can be mitigated by using tumor-informed ctDNA assays, paired tumor–normal sequencing, evaluation of variant allele frequencies and gene context typical of CHIP, and correlating molecular findings with tissue genomics and clinical behavior over time.

The literature agrees that MUC-RP prognosis is poor, with most patients living between 2 and 5 years from the time of early diagnosis (4). There have been minimal reports of patients receiving postoperative treatment on advanced disease with unclear survival outcome (4). One case report by Raphael et al. (2011) (18) involved the administration of radiotherapy after surgical resection of MUC-RP, with recurrence at 1 year later in the renal fossa, enlarged retroperitoneal lymph nodes, and diffuse metastases to the cervical and lumbar spine. In a report by Higgins et al. (2017) (19), capecitabine was utilized for adjuvant therapy to treat MUC-RP within a mature cystic teratoma, modeled after treatment for pancreatic mucinous adenocarcinoma; however, patient outcomes or survival were not reported. Giannakodimos et al. (2025) (20) reported primary adenocarcinoma of the upper urinary tract in 55 cases with 6- and 12-month cumulative survival rates of 18.05% (95% CI: 9.7%–35.61%) and 25.20% (95% CI: 14.55%–43.62%), respectively, with papillary and tubulovillous subtypes presenting with a moderate prognosis, while the mucinous and poorly differentiated subtypes had a significantly poor prognosis (HR: 91.06, 95% CI: 7.31–1,134.32, *p* = 0.002). Our patient has remained alive for more than 3 years after her diagnosis, perhaps due to a more aggressive surgical, antineoplastic, and supportive care approach.

### Strengths and limitations

This case report presents the first comprehensive molecular profiling of MAC-RP with XGP, providing new insights into its genetic makeup and clinical behavior. Our findings highlight the importance of incorporating tumor-specific biomarkers, along with serial ctDNA and cfDNA monitoring, to guide treatment choices and predict disease progression. By showing that MAC-RP may be more closely related to urothelial rather than colorectal cancers at the genomic level, our study supports customizing treatment based on gene expression origin rather than histologic appearance. This precision medicine approach could help improve outcomes in this rare and aggressive cancer, which currently has no established guidelines. As an inherently limited single-case report, these findings should be interpreted cautiously; however, the use of novel molecular techniques in this rare and extreme case mainly demonstrates future potential clinical applications, aligned with emerging evidence-based research rather than drawing general conclusions.

### Patient’s perspective

“Learning more about my own case through this process was deeply meaningful to me, and I am grateful that my experience may help others with rare tumors in the future. I only wish this kind of research had been available earlier, and I hope sharing my story encourages continued study to benefit patients who come after me.”

## Data Availability

The datasets presented in this article are not readily available because Patient’s information. Requests to access the datasets should be directed to megant6139@yahoo.com.
